# Supplementary material for: A Case Study in Saudi Arabia: Biodiversity of Maize Seed-Borne Pathogenic Fungi in Relation to Biochemical, Physiological, and Molecular Characteristics
Source: Plants (Basel). 2022 Mar 21;11(6):829. doi: 10.3390/plants11060829 (PMC8954539; doi:10.3390/plants11060829)
Supplement: Supplementary file 1 [file plants-11-00829-s001.zip › plants-1626400-supplementary.pdf]

A case study in Saudi Arabia: biodiversity of maize seed-borne pathogenic fungi in relation to biochemical, physiological, and molecular characteristics

Abdulaziz A. Al-Askar [1*], Khalid M. Ghoneem [2], Elsayed E. Hafez [3] and WesamEldin I. A. Saber [4*]

1 Botany and Microbiology Department, Faculty of Science, King Saud University, Riyadh 11451, Saudi Arabia.

2 Seed Pathology Research Department, Plant Pathology Research Institute, Agricultural Research Center (ID: 60019332), Giza 12619, Egypt.

3 Plant Protection and Biomolecular Diagnosis Department, Arid Lands Cultivation Research Institute, City of Scientific Research and Technological Applications, New Borg El-Arab, Alexandria 21934, Egypt.

4 Microbial Activity Unit, Microbiology Department, Soils, Water and Environment Research Institute, Agricultural Research Center (ID: 60019332), Giza 12619, Egypt.

* Correspondence: aalaskara@ksu.edu.sa (A.A.A.-A); wesameldin.saber@arc.sci.eg; Tel.: +20-111-173-1062

Table S1. Sequences of ITS and RAPD primers that were used in this study.

Primer name	RAPD Sequence (5→3̀)	Annealing temperature
ITS 1	TCTGTAGGTGAACCTGCGG	51 ºC
ITS 4	TCCTCCGCTTATTGATATGC	
RAPD1	CAGCACCCAC	
RAPD2	GGGTAACGCC	37 ºC
RAPD3	CATCCCCCTG	
RAPD4	CTCACCGTCC	
RAPD5	GACGGATCAG	
RAPD6	CACGAGTCTC	
RAPD7	CCCTACCGAC	

Table S2. GeneBank accession number, and morphological identification of phytopathogenic fungal strains isolated from maize grains.

Fungus	GeneBank accession number	Colony morphology	Conidiophores	Conidia
Alternaria alternata KSU4M1-1	**MW553174**	On PDA, colonies are filamentous, growing fast, grey, dark brown, or black.	Conidiophores are single or in small groups, straight or curved, sometimes geniculate, 13–59.5 µm × 2-4.1 µm, with scars.	Conidia are ellipsoidal, ovoid, obclavate, obpyriform in chains, 9–13 µm × 20–36 µm in size, often with a short conical or cylindrical beak 1.7–9.2 µm in diameter tapering to the apex or blunt, about one third or one-quarter of the conidial length. Body with 1–4 transverse septa and 0–2 longitudinal septa, sometimes oblique, in each cell, golden-brown to brown, beak paler.
Curvularia lunata KSU3M1-1	**MW422773**	On PDA, colonies effuse brown, blackish-brown or black, hairy, or velvety. Mycelium is mostly immersed.	Conidiophores are solitary or in small groups, simple or branched, straight or flexuous, pale to dark brown, septate, up to 650µ long, 5-9µ thick often swollen at the base 10-15µ.	Conidia acropleurogenous, 3 septate, almost always curved at the third cell from the base which is usually longer and often darker than the others, cell at each end subhyaline or pale brown, intermediate cells mid to dark brown, smooth, 20-32 × 9-15µm.

Curvularia australiensis KSU3M2-1	MW553173	On PDA, colonies effuse blackish-brown with light gray aerial hyphae, velvety. Hyphae pale to mid-brown, smooth, septate	Conidiophores are single, flexuous, geniculate, septate, smooth, cylindrical, reddish-brown, up to 235 × 6-15 µm. Conidiogenous cells with verruculose nodes.	Conidia straight, ellipsoidal, or oblong, rounded at the ends, reddish-brown to black-brown, usually 3 or 4-5-distoseptate, 27-43 × 7.5-10 µm, 32 × 9 µm on average.
Fusarium chlamydosporum KSU1M3-1	MW422770	Rapid growing on PDA, reaching 5.5 cm diam in 4 days at 25°C, abundantly developed pink aerial mycelium. Sprodochia are absent. Undersurface is red-vinaceous.	Scattered over the aerial mycelium, richly branched and unbranched polyphialides and monophilides	Microconidia abundant, spindle-shape, 0-1 septate, borne usually on polyphilides, fusiform or elongate, 8.5-9.2×2.7-3.0 µm. They have a thick wall, and moderately curved. Macroconidia not detected. Chlamydospores numerous, intercalary, roughened, 8.7-16 µm.
Fusarium proliferatum KSU1M2-2	MW405882	Rapid growing on PDA, with purple-violet aerial mycelium with age. Sprodochia, absent. The undersurface is generally purple.	Unbranched and branched polyphialides and monophilides	Microconidia are abundant, single-celled, clavate, or pyriform, forming on a long chain and in false heads, 7.6-8.8×2.6-3.1 µm. Macroconidia, rarely produced, almost straight, 47-54 × 3.1-3.5µm. The walls are thin and delicate, and the basal-cell is foot-shaped. Chlamydospores absent.

Fusarium proliferatum KSU1M2-3	**MW405883**	Rapid growing on PDA, with dense aerial mycelium that is white at first and becomes tan to brown as the culture ages. Orange Sprodochia appears as the culture ages. Undersurface is initial peach color changes to dark brown.	Unbranched and branched polyphialides and monophilides.	Macroconidia are only produced, falcate, have 4-7 distinct septa, with a well-developed pedicellate foot cell and attenuated apical cell which curved inwards, 45-52 × 3.7-4.1 µm diameter. Chlamydospores abundant, intercalary, solitary, in chain or knots, globose, 6.7-8.6 µm diam.
Fusarium verticillioides KSU1M1-1	**MW405871**	Rapid growth on PDA, with white aerial mycelium and becomes tinged with purple, 4 cm diam in 4 days at 25° C. Orange Sporodochia were present as a pseudo-pionnotal mass. Undersurface is generally dark purple.	Unbranched and branched monophilides, which are occasionally produced in pairs to give a rabbit-ear appearance.	Microconidia are abundant, single-celled, 0 to 1-septate, oval to club-shaped, 7–9.7 x 2.5-3.0 µm, and have a flattened base. They are formed in long chains and false heads. Macroconidia sparse, slightly sickle-shaped to almost straight, 3-5 septa, 36–57 x 2.9-3.6 µm. Apical cells are curved and often tapered to a point, while basal cells are notched or foot-shaped. Chlamydoconidia absent

Fusarium verticillioides KSU1M1-2	**MW405884**	Rapid growth on PDA, with white aerial mycelium and becomes tinged with purple, 4 cm diam in 4 days at 25° C. Orange Sporodochia were present as a pseudo-pionnotal mass. Undersurface is generally dark purple.	Unbranched and branched monophilides, which are occasionally produced in pairs to give a rabbit-ear appearance.	Microconidia are abundant, single-celled, 0 to 1-septate, oval to club-shaped, 7.2 –9.2 x 2.6-3.2 µm, and have a flattened base. They are formed in long chains and false heads. Macroconidia sparse, slightly sickle-shaped to almost straight, 3-5 septa, 35-55 × 3-3.6 µm. Apical cells are curved and often tapered to a point, while basal cells are notched or foot-shaped. Chlamydoconidia absent
Fusarium verticillioides KSU1M1-3	**MW405885**	Rapid growth on PDA, with white aerial mycelium and becomes tinged with purple, 4 cm diam in 4 days at 25° C. Orange Sporodochia were present as a pseudo-pionnotal mass. Undersurface is generally dark purple.	Unbranched and branched monophilides, which are occasionally produced in pairs to give a rabbit-ear appearance.	Microconidia are abundant, single-celled, 0 to 1-septate, oval to club-shaped, 7.4 –9.5 × 2.3-3.3 µm, and have a flattened base. They are formed in long chains and false heads. Macroconidia sparse, slightly sickle-shaped to almost straight, 3-5 septa, 39–57 × 2.9-3.6 µm. Apical cells are curved and often tapered to a point, while basal cells are notched or foot-shaped. Chlamydoconidia absent

Fusarium verticillioides KSU1M1-4	**MW422779**	Rapid growth on PDA, with white aerial mycelium and becomes tinged with purple, 4 cm diam in 4 days at 25° C. Orange Sporodochia were present as a pseudo-pionnotal mass. Undersurface is generally dark purple.	Unbranched and branched monophilides, which are occasionally produced in pairs to give a rabbit-ear appearance.	Microconidia are abundant, single-celled, 0 to 1-septate, oval to club-shaped, 6.9 –9.7 × 2.7-3.1 µm, and have a flattened base. They are formed in long chains and false heads. Macroconidia sparse, slightly sickle-shaped to almost straight, 3-5 septa, 34–58 × 3.2-3.7 µm. Apical cells are curved and often tapered to a point, while basal cells are notched or foot-shaped. Chlamydoconidia absent
Sarocladium zeae KSU2M2-1	**MW422774**	Colony growth is slow, flat to very thin cottony, white to pink, reaching 1.8-2.5 cm diameter after 10 days in the dark at 20°C; thin hyphae, producing awl-shaped and erect phialides with a septum at the base, formed singly or in very simple branching conidiophores.	Conidiophores usually several times branched, with phialides, up to 80 µm, chromophilic at the base, arranged in whorls, and conidia in slimy heads; sclerotia and chlamydospores absent.	Conidia unicellular, cylindrical with rounded ends, smooth, hyaline, in slimy heads, mostly 3.2-5.4 × 0.9-1.7 µm.

***Sarocladium zeae* KSU2M2-2**	**MW422772**	Colony growth is slow, flat to very thin cottony, white to pink, reaching 1.7-2.4 cm diameter after 10 days in the dark at 20°C; thin hyphae, producing awl-shaped and erect phialides with a septum at the base, formed singly or in very simple branching conidiophores.	Conidiophores usually several times branched, with phialides, up to 80 µm, chromophilic at the base, arranged in whorls, and conidia in slimy heads; sclerotia and chlamydospores absent.	Conidia unicellular, cylindrical with rounded ends, smooth, hyaline, in slimy heads, mostly 3.1-5.3 × 0.9-1.8 µm

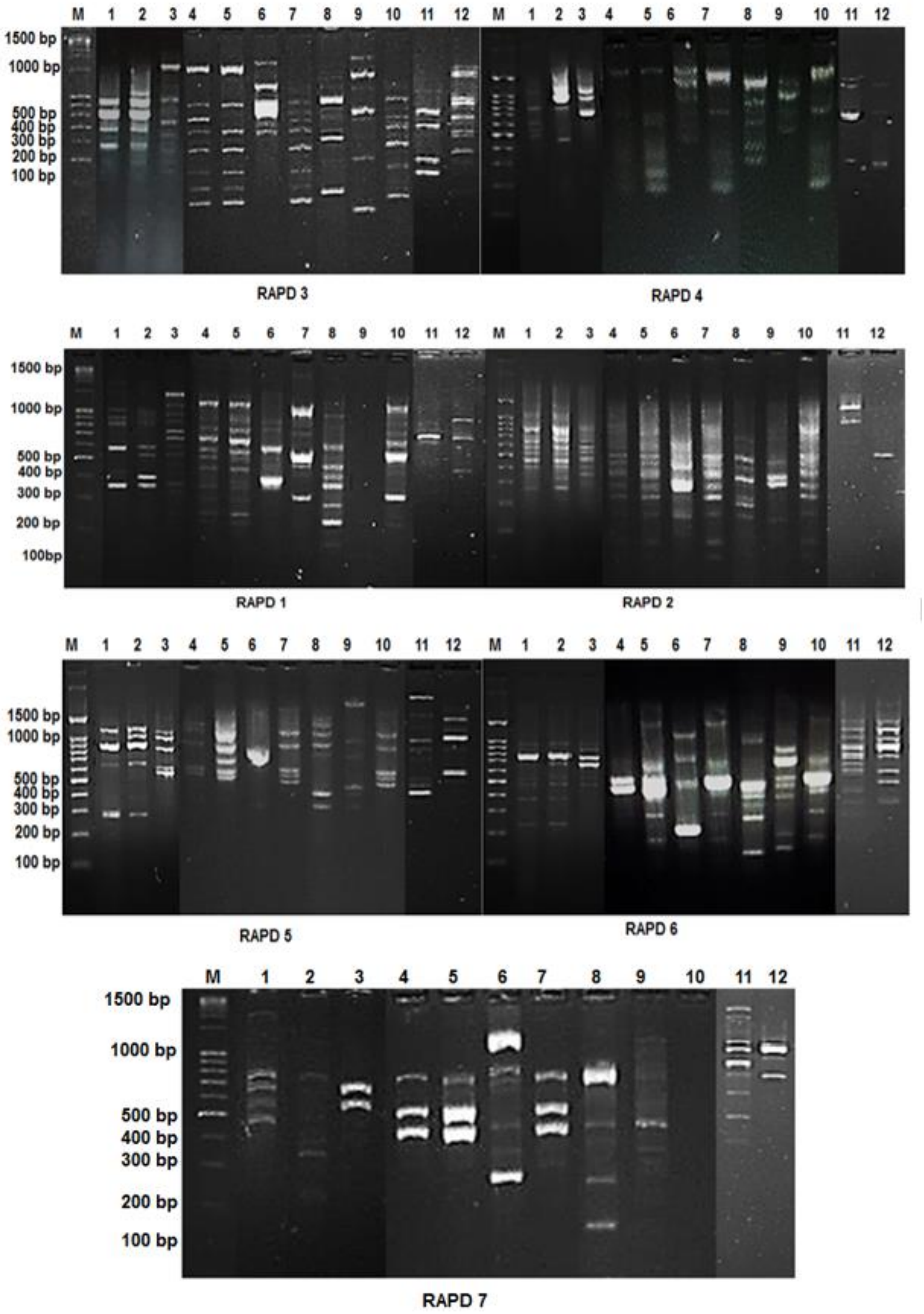

Figure S1. Intra-genotyping among the 12 fungal isolates using RAPD PCR with 7 different arbitrary primers. Lanes; M: 100 bp DNA marker, 1-12 the fungal isolates in sequence; 1: KSU4M1-1; 2: KSU3M2-1; 3: KSU3M1-1; 4: KSU1M3-1; 5: KSU1M2-2; 6:

KSU1M2-3; 7: KSU1M1-1; 8: KSU1M1-2; 9: KSU1M1-3; 10: KSU1M1-4; 11: KSU2M2-1; 12: KSU2M2-2.
